# IDR knowledge base for primary immunodeficiencies

**DOI:** 10.1186/1745-7580-3-6

**Published:** 2007-03-29

**Authors:** Crina Samarghitean, Jouni Väliaho, Mauno Vihinen

**Affiliations:** 1 Institute of Medical Technology, FI-33014 University of Tampere, Finland; 2 Research Unit, Tampere University Hospital, FI-33520 Tampere, Finland

## Abstract

**Background:**

The ImmunoDeficiency Resource (IDR) is a knowledge base for the integration of the clinical, biochemical, genetic, genomic, proteomic, structural, and computational data of primary immunodeficiencies. The need for the IDR arises from the lack of structured and systematic information about primary immunodeficiencies on the Internet, and from the lack of a common platform which enables doctors, researchers, students, nurses and patients to find out validated information about these diseases.

**Description:**

The IDR knowledge base, first released in 1999, has grown substantially. It contains information for 158 diseases, both from a clinical as well as molecular point of view. The database and the user interface have been reformatted. This new IDR release has a richer and more complete breadth, depth and scope. The service provides the most complete and up-to-date dataset. The IDR has been integrated with several internal and external databases and services. The contents of the IDR are validated and selected for different types of users (doctors, nurses, researchers and students, as well as patients and their families). The search engine has been improved and allows either a detailed or a broad search from a simple user interface.

**Conclusion:**

The IDR is the first knowledge base specifically designed to capture in a systematic and validated way both clinical and molecular information for primary immunodeficiencies. The service is freely available at http://bioinf.uta.fi/idr and is regularly updated. The IDR facilitates primary immunodeficiencies informatics and helps to parameterise *in silico *modelling of these diseases. The IDR is useful also as an advanced education tool for medical students, and physicians.

## Background

Primary immunodeficiency disorders (PIDs) impair the function of the immune system. Patients with these intrinsic defects have increased susceptibility to recurrent and persistent infections, and they may also have autoimmune and cancer related symptoms. Most PIDs are rare and the diagnosed patients for a condition are often randomly spread out around the world. More than 150 PIDs affecting the immune system have been described and more than 100 genes involved in PIDs have been identified [1]. The number of mutations, identified in unrelated families with different PIDs, totals over 4,500 [2].

There is plenty of information related to immunology and immunodeficiencies on the Internet. General immunome information can be found e.g. from IMGT [3], AntiJen [4] and Immunome [5] databases and more specific data e.g. in ImmTree [6], IDbases [2], SYFPEITHI [7], and Immune Epitope Databases and Analyse Resources (IEDB) [8]. The scattering of the disease-related information in the literature and the Internet is a big obstacle for those interested in rare diseases. Users often have problems in finding relevant information and assessing the quality of information from the Internet. Biomedical information holds promises for developing informatics methods for postgenomic and personalised medicine. The new knowledge can be applied in the prevention, diagnosis and treatment of diseases. Computerised information sources have many challenges related, for example, to terminology and ontology building, information extraction from texts, knowledge discovery from collections of documents, sharing and integrating knowledge from factual and textual databases, and semantic annotation. There is a need for a standardised nomenclature and data form that can be easily handled by computers and presented on any platform.

The ImmunoDeficiency Resource (IDR) integrates biomedical information related to PIDs into a web accessible knowledge base. The fact files, which form the core of the system, integrate biomedical knowledge from several heterogeneous and autonomous sources.

This paper illustrates numerous new features and improvements, which have been implemented since previous IDR releases [9,10], and details about data collection and automated database integration. The IDR is developed to serve anybody interested in PIDs and to provide relevant, up-to-date and validated information in an easily understandable and usable format.

## Construction and Content

The IDR has been designed and implemented using eXtensible Markup Language XML [11], a system comprising a native XML database and an XML server. Data within the IDR is structured into document-centred XML and SHTML files. The interface to the IDR has been completely redesigned. It consists of a dynamic layout that can adapt to different screen sizes, from wide desktop screens to small mobile devices.

Numerous new features, such as the classification of PIDs, genes related to immunodeficiency, reference sequences, protein structures and animal model pages, have been added. Links are also provided to other IDR-fact file databases [12], IDbases for PID-causing mutations [2], and IDdiagnostics for PID diagnostic laboratories [13].

**Figure 1:**
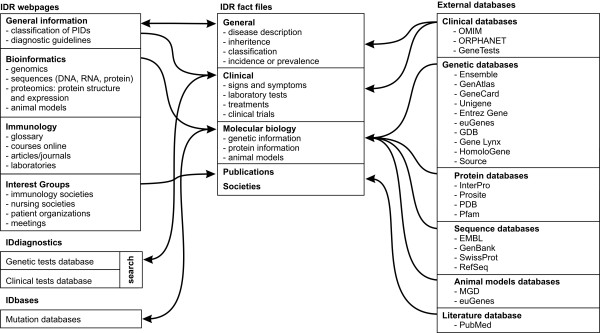
**Concept map for the IDR knowledge service**. IDR is composed of web pages grouped in to different class categories and a fact file database. The system is integrated with different internal and external databases to serve a wide category of users.

The IDR aims to provide comprehensive integrated knowledge about immunodeficiencies in an easily accessible form, targeting different types of users (doctors, scientists, nurses and patients and their families). The resource includes clinical, biochemical, genetic, structural and computational data and analyses. The main headings of the IDR are General Information, Bioinformatics, Immunology, and Interest Groups (Fig. 1).

### The General Information class

Immunodeficiencies in the IDR are classified according to the molecular defects criteria [1] with links to the Online Mendelian Inheritance in Man (OMIM) database [14]. Information about the affected genes and loci are provided and linked with corresponding services. The ESID and PAGID recommendations for diagnostic criteria [15], the American Academy of Allergy, Asthma and Immunology (AAAAI) parameters [16], and different diagnostic guidelines [17] are also included. There is also a list for PID abbreviations.

**Figure 2:**
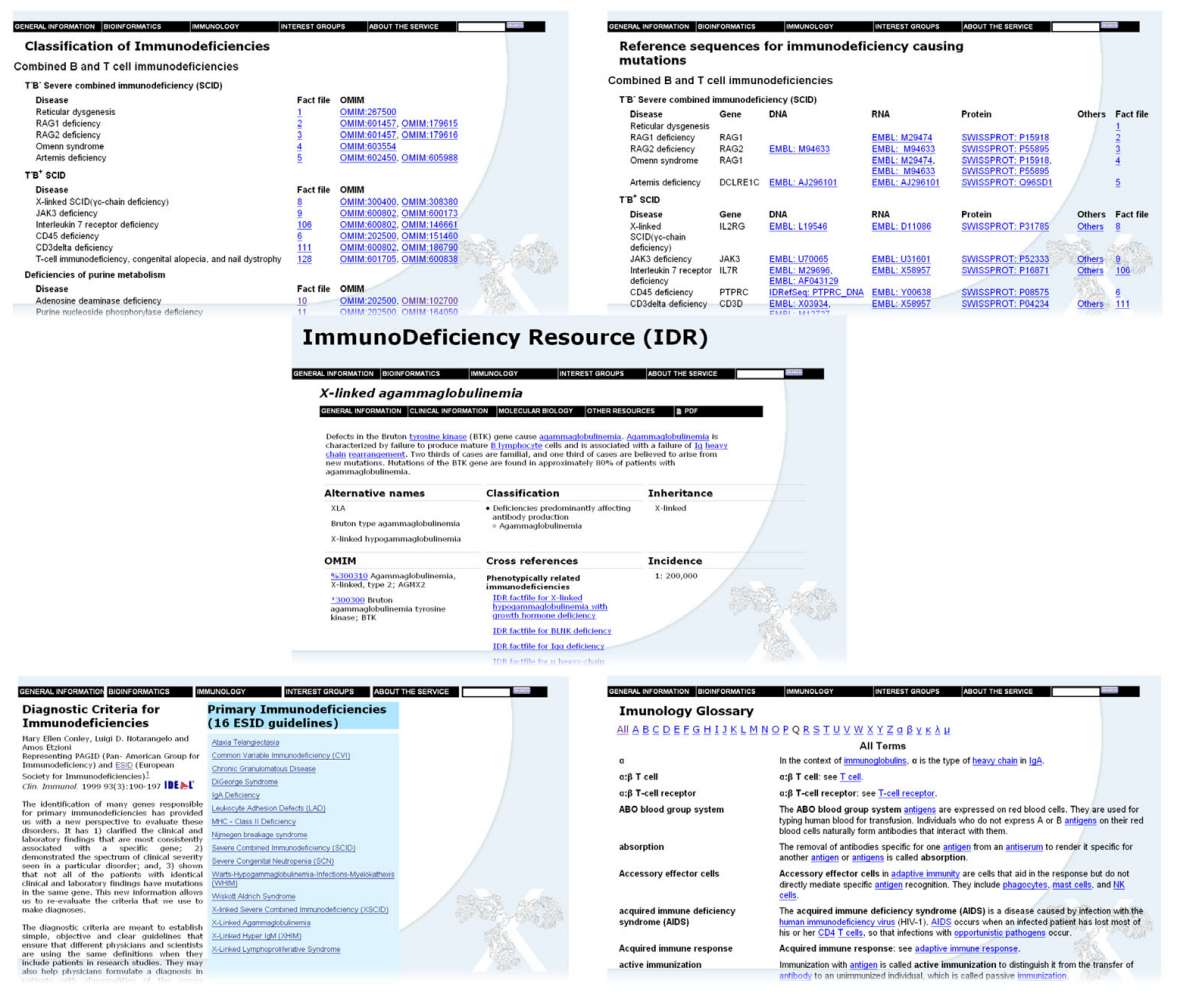
**IDR user interface**. Screenshots of the main IDR pages. The new user interface provides faster access to the information and different new features, such as classification of diseases (top left), the gene related with the PIDs and their reference sequences (top right), glossary terms for immunology (bottom right), and diagnostic tools (bottom left). At the core of the system are fact files that provide clinical and molecular information for 158 primary immunodeficiency diseases (centre).

At the core of the system are fact files (Fig. 2), which store information regarding disorders, genes, mutations, protein sequences, online resources, organisations and associations [12]. At present there are fact files for 158 diseases. The user interface allows fast access to the information. International Classification of Diseases (ICD) codes [18] are provided for those diseases where the codes are available. Each fact file provides basic information about the disease and the affected gene. The fact files have hyperlinks to other reliable Internet resources. The fact file data model and the Inherited Disease Markup Language (IDML) [12] were developed to facilitate disease information integration, storage and exchange. The fact files make use of the following specifications, standards and databases: HUGO nomenclature [19], Swiss-Prot [20], GeneCard [21], and SOURCE [22].

The IDML fact files have been generated for each PID. The major concepts in the fact files are general information, clinical information, molecular biology and other resources – all of which are linked to related information services (Fig. 1). Each of these elements comprises one or more additional levels. Table 1 summarised the major concepts and descriptions of the elements in the IDR-fact files.

**Table 1:** Major concepts and elements in IDR-fact files

**Major concepts**	**Elements**	**Description**
General Information	DiseaseName	Disease name
	Abbreviation	Abbreviation for disease name
	AlternativeNames	Alternatively used disease names
	Description	General description of disease
	Classification	Classifies disease in the fact files' hierarchy
	Omim	Link to the OMIM knowledge base
	ICD-10	WHO classification of diseases
	CrossReferences	References to the related fact files
	Incidence	Number of cases in population

Clinical Information	Clinical Description	Characteristic clinical features
	Diagnosis	Diagnostic guidelines, protocols and laboratories
	TherapeuticOptions	Treatment of disease
	ResearchPrograms	Clinical trials or research projects on-going

Molecular Biology	GeneInformation	Gene name, aliases, reference sequences, chromosomal location, maps, markers, variations and other gene related resources
	AnimalModels	Related transgenic animal data
	ProteinInformation	Protein features, structures, domains, motifs and other protein resources
	ExpressionPattern	Gene expression levels in a variety of cells and tissues

Other Resources	Publications	Related publications in PubMed
	Societies	General and disease specific societies
	OtherSites	Other related websites

The IDML schema [23], IDML document type definition file [24], examples of an IDML-document, and documentation on the syntax can be read from [25]. The validation in IDML fact files is done with the IDML validator program, available online at [26].

### The Bioinformatics class

The bioinformatics section integrates numerous Web based services (Ensemble [27], Source [22], EntrezGene [28], euGenes [29], GeneLynx [30], UniGene [31], GeneCard [21], GenAtlas [32]). Reference sequences for PID genes are available for DNA and RNA from the EMBL database, and for protein data from SwissProt [20]. When available, there are links to the protein structures and visualisation tools in the PDB [33]. The animal models page has been updated.

The IDbases [2] section provides, in addition to our own mutation registries, links to other IDbases. At the moment we have 115 databases with over 4,500 patient entries.

### The Immunology and Interest Group classes

The immunology section lists collections of immunology related data sources including lectures on immunology and immunodeficiencies, and links to over 40 online immunology journals. A new feature is the glossary, which provides explanations for more than 800 immunology terms. Glossary terms are cross linked by each other, so by clicking one of these terms, such as 'antigen', not only is the explanation for the term provided, but also for a group of terms related to 'antigen'. This gives a broad overview of immunology terminology, which also makes it a useful tool for education.

The interest group section contains links to immunology, immunodeficiency, and nursing and patient organisations. Several societies are related to immunodeficiency research, care and patients. The list of meetings and workshops is continuously updated.

## Utility and Discussion

### Accuracy and validation of data

The Internet contains a large number of pages. Search engines often give thousands of links but usually the most difficult task is to differentiate the useful and reliable data from other search results. In the IDR, the experts check all the data and approve only those sites with solid scientific and medical information. There will be at least one external expert for each immunodeficiency. Nursing and patient societies are also involved in the data validation process for their own interest groups.

The IDR is easy to navigate. The pages are colour coded for different interest groups: researchers, physicians, nurses, patients and families. By selecting the group of interest, the user can get specific pages produced and tailored for the particular group. This makes it easier for the user to find the most relevant and useful information. The IDR also provides an advanced text search facility, which can utilise Boolean logic searches with multiple keywords. Within a typical search, the user-entered search criteria are carried from an SHTML or XML form to a category specific PERL script, which performs the database queries.

The IDR can be used to discover many kinds of information as it is integrated with internal (IDbases and IDdiagnostics) and external databases (Fig. 1).

The IDbases have recently been integrated with the ESID patient registry [34], which collects clinical data for patients. This collaboration facilitates direct submission both to the ESID registry and IDbases. A similar arrangement will be made with the US Immunodeficiency Network (USIDNET).

A fact file is a user oriented user interface, which serves as a good starting point to explore information on hereditary diseases. The user can find not only information about the disease nomenclature and classification (OMIM, ICD10), but also a clinical description of the disease, inheritance and prevalence. The IDR-fact file facilitates finding information about laboratories which perform genetic tests for PIDs, using direct links to IDdiagnostics [13], GeneTest [35] or ORPHANET [36]. Information for PID related genes contains the nomenclature, including aliases, sequences, chromosomal location and maps, variations, and mutations. The IDR-fact files also contain information about protein functions, structure, domains, motifs, subcellular location and post-translational modifications.

PID researchers will also find in the IDR-fact files lots of information for each disease to keep up-to-date with the literature, meetings and different associations in the field.

### Future work

In the near future a new tool for faster and more accurate diagnosis, PIDexpert, will be added. PIDexpert is a medical expert system, designed to give the diagnostic picture of PIDs based on symptoms, signs, medical history, physical findings, and laboratory tests. Future tasks in the development of the IDR will focus on expanding its depth, breadth, and scope. The external database updates will be monitored so that any alterations are mirrored within the archive. We plan to further develop the IDR based on user feedback and our interactions with the PID community.

## Conclusion

The IDR contains systematically organised, continuously updated and validated information that is valuable for clinicians and researchers and can improve the medical care of PIDs. IDR is the first knowledge service designed to capture both clinical and molecular information about primary immunodeficiencies and to address different types of users. It is validated and will be updated frequently. The IDR facilitates PID informatics and helps to parameterise *in silico *modelling of these diseases. The IDR has many potential users throughout the PID community, from doctors to patients and from immunoinformaticians to experimental immunologists and structural biologists.

## Availability and requirements

The IDR database is freely available for academic use from the URL: .

## Competing interests

The author(s) declare that they have no competing interests.

## Authors' contributions

CS carried out the data mining analysis, database development and drafted the manuscript. JV participated in database development and drafted the manuscript. MV conceived the study, participated in IDR design and coordination, and drafted the manuscript. All authors read and approved the final manuscript.
